# Determining Molecular-Level Interactions of Carboxyl-Functionalized Nanodiamonds with Bacterial Membrane Models as the Basis for Antimicrobial Activity

**DOI:** 10.1021/acs.langmuir.4c05173

**Published:** 2025-03-02

**Authors:** Giovanna Eller Silva Sousa, Bruna Alves Martins, Alexandre Mendes de Almeida Junior, Rafaela Campos Queiroz, Dayane Batista Tada, Sabrina Aléssio Camacho, Osvaldo N. Oliveira Jr., Pedro Henrique Benites Aoki

**Affiliations:** † School of Sciences, Humanities and Languages, São Paulo State University (UNESP), Assis, SP 19806-900, Brazil; ‡ Institute of Science and Technology, Federal University of São Paulo (UNIFESP), São José dos Campos, SP 12231-280, Brazil; § São Carlos Institute of Physics, University of Sao Paulo (USP), São Carlos, SP 13566-590, Brazil

## Abstract

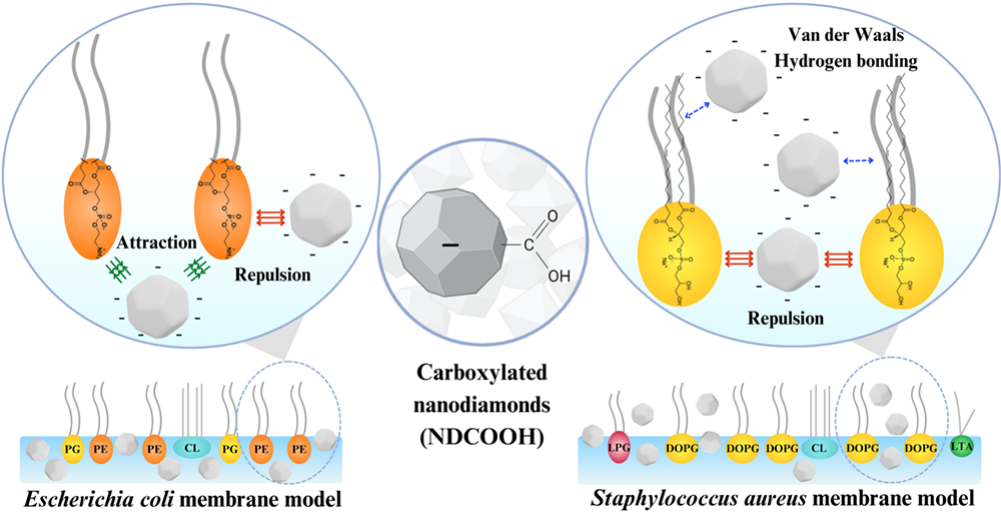

Carbon-based nanostructures, such as carboxylated nanodiamonds (NDCOOHs), are promising to combat resistant bacterial strains by targeting their protective membranes. Understanding their interactions with bacterial membranes is therefore important for elucidating the mechanisms underlying NDCOOHs antimicrobial activity. In this study, we investigated the incorporation of NDCOOHs into lipid Langmuir monolayers mimicking cytoplasmic membranes of **Escherichia coli** and *Staphylococcus aureus*, model systems for Gram-negative and Gram-positive bacteria, respectively. Using polarization-modulated infrared reflection–absorption spectroscopy (PM-IRRAS), we observed significant interactions between NDCOOHs and the polar head groups of the **E. coli** lipid monolayer, driven by electrostatic attraction to ammonium groups and repulsion from phosphate and carbonyl ester groups, limiting deeper penetration into the lipid chains. In contrast, *S. aureus* monolayers exhibited more pronounced changes in their hydrocarbon chains, indicating deeper NDCOOHs penetration. NDCOOHs incorporation increased the surface area of the **E. coli** monolayer by approximately 4% and reduced that of *S. aureus* by about 8%, changes likely attributed to lipid oxidation induced by superoxide and/or hydroxyl radicals generated by NDCOOHs. These findings highlight the distinct interactions of NDCOOHs with Gram-positive and Gram-negative lipid membranes, offering valuable insights for their development as targeted antimicrobial agents.

## Introduction

Bacterial infections represent a challenge for public health on a global scale.^1^ Pathogenic microorganisms can cause a wide range of infections in humans, from mild illnesses to severe conditions that may, in some cases, be fatal.^2,3^ The spread of antibiotic-resistant bacterial strains has significantly worsened this scenario, making conventional treatments less effective.^4^ The structural diversity of bacterial cell membranes can be classified using Gram staining, which highlights the differences between Gram-positive and Gram-negative bacteria.^5^ Gram-positive bacteria possess a single lipid membrane followed by a thick peptidoglycan layer, whereas Gram-negative bacteria feature two lipid membranes separated by a peptidoglycan layer.^5^ As the primary barrier against external substances, the bacterial membrane is essential in mediating interactions with antimicrobial agents and other external compounds. Despite differences in membrane architecture, both Gram-positive and Gram-negative bacteria rely on their membranes as key defense mechanisms.^6^ Gram-negative bacteria utilize outer membrane porins for the passage of small molecules, including hydrophilic antibiotics, though mutations in these proteins can confer resistance.^7^ Conversely, Gram-positive bacteria strengthen their cell walls or produce enzymes that degrade antibiotics, highlighting the diverse mechanisms underlying bacterial resistance.^6^

The rise of super-resistant bacteria has outpaced the development of antibiotics, thus creating the need for alternative treatments.^7^ A promising strategy involves the use of nanomaterials with antibacterial properties,^8−11^ particularly carbon-based nanostructures, such as carbon nanotubes, nanofibers, fullerenes, and diamond nanoparticles (NDs).^10^ NDs, made of carbon atoms in a diamond cubic crystal structure with sp^3^ hybridization, can be synthesized through various methods,^12−23^ which impart not only the typical properties of diamonds but also unique nanomaterial characteristics, such as fluorescence, biocompatibility, possible surface modification, and antimicrobial activity.^8−11,23−25^ The antimicrobial effectiveness of NDs depends on their surface chemical groups, which can vary depending on synthesis conditions, processing, and functionalization^24,25^ and affect the colloidal stability of NDs suspensions. Functionalization enables attachment of diverse surface chemical groups, such as carboxyl, hydroxyl, amino, sulfur, ester, and anhydride.^13^ Functionalizing nanodiamonds with carboxyl groups to produce NDCOOHs has demonstrated strong antimicrobial activity, inducing cell death in **Escherichia coli**, **Bacillus subtilis**, *Staphylococcus epidermidis* and *Staphylococcus aureus* cultures.^9,11,26−30^ The antibacterial effects are thought to result from interactions with cellular components, including direct physical disruption of the bacterial surface or interactions with the reactive nanostructure surface, leading to defects in the cell envelope.^9,10,31^ Determining the interactions with the lipid membrane, in particular, may help understanding the antimicrobial mechanisms of NDCOOHs. However, such studies involving NDs are scarce in the literature. Furthermore, the bactericidal activity of NDCOOHs is influenced by proteins or other components in the cell medium, complicating efforts to isolate and study their localized effects on the cell membrane, especially lipid-nanoparticle interactions.^32,33^ To address these challenges, suitable methods are needed to both mimic the cell membrane and provide detailed molecular insights into NDCOOHs interactions.

Langmuir monolayers are recognized as effective models for studying bacterial membranes, providing valuable insights into the mechanisms of action of biologically relevant materials.^34−41^ However, most studies in the literature rely on simplified lipid compositions, often limited to single lipids or binary mixtures. For instance, Casas-Sanchez et al.^42^ demonstrated stronger interactions of oleuropein with PG monolayers, a model for *S. aureus*, compared to PE monolayers, correlating with its higher activity against Gram-positive bacteria. Barbosa et al.^43^ showed that Labaditin interacts with PG/CL mixtures mimicking Gram-positive membranes, forming pore-inducing nanotubes, while exhibiting limited interaction with LPS monolayers representing Gram-negative outer membranes.^44^ Similarly, Wölk et al.^45^ and Martins et al.^46^ used DPPG-based and PG/CL monolayers to investigate lipid-surfactant miscibility and β-lactam resistance, respectively. To the best of our knowledge, the only studies involving nanodiamonds were presented by Chakraborty et al.,^47,48^ which focused on human cell membrane models rather than bacterial systems. Their findings showed that the lipid headgroup charge and alkyl chain saturation affect domain organization in monolayers, with electrostatic repulsion driving domain packing changes in anionic phospholipid systems and mixed monolayers with excess zwitterionic lipids. The anionic nanodiamonds enhanced material retention and reincorporation in systems containing negatively charged lipids but caused material loss in unsaturated lipid mixtures.

In this study, we used Langmuir monolayers as biomimetic models of **E. coli** and *S. aureus* membranes to investigate molecular-level interactions with NDCOOHs. In contrast to studies that often use simplified lipid compositions, we employed rather complex systems to better mimic bacterial cell envelopes. The **E. coli** model was based on lipid extracts, mimicking the inner membrane, while the *S. aureus* model used a mixture of 75% dioleoylphosphatidylglycerol (DOPG), 15% lysylphosphatidylglycerol (LPG), 3% cardiolipin (CL), and 7% lipoteichoic acid (LTA). Surface pressure–area (π–*A*) isotherms assessed monolayer behavior and NDCOOHs adsorption efficiency, while polarization-modulated infrared reflection absorption spectroscopy (PM-IRRAS) analyzed lipid orientation and structural changes.

## Experimental Section

### Materials and Solutions

**Figure 1:**
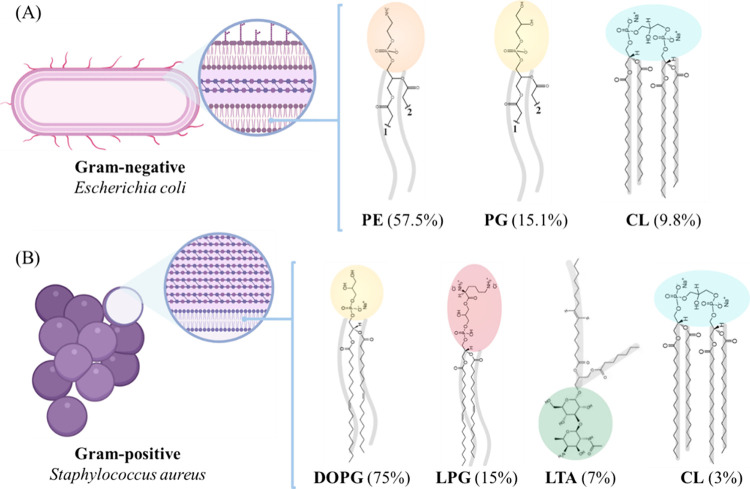
Schematic illustration for (A) **E. coli** (Gram-negative) and (B) *S. aureus* (Gram-positive) bacteria along with their respective membrane structures (insets). The lipid membrane regions mimicked in this study are indicated, and the molecular structures of the lipids in the composition of (A) **E. coli** lipid extract and (B) *S. aureus* lipid mixture are shown. The numbers 1 and 2 refer to the lipid chains of PE and PG, which can have different sizes and be unsaturated or not.

**Escherichia coli* (*E. coli*)* total lipid extract (strain B; ATCC 11303; Avanti Polar Lipids, 100500P) consists of 57.5% phosphatidylethanolamines (PE), 15.1% phosphatidylglycerols (PG), 9.8% cardiolipins (CL), and 17.6% unidentified components. The **E. coli** lipid extract solution was prepared in chloroform at a concentration of 1 g/L. The lipid membrane of *Staphylococcus aureus* (*S. aureus*) was mimicked following compositions suggested in the literature, predominantly consisting of PG, lysylphosphatidylglycerols (LPG), and CL.^49−51^ The lipid mixture was prepared by combining 75% dioleoylphosphatidylglycerol (DOPG), 15% LPG, 3% CL (Avanti Polar Lipids, 840475P, 840521P and 710335, respectively), and 7% lipoteichoic acid from *S. aureus* (LTA) (Sigma-Aldrich, L2515). These lipids were solubilized in chloroform at a concentration of 1 mmol/L. The molecular structures of the lipids in the composition of **E. coli** lipid extract and *S. aureus* lipid mixture are shown in Figure 1a,b, respectively.

Phosphate-buffered saline (PBS, pH = 7.4, Sigma-Aldrich, P4417) solution was prepared by dissolving the powder in ultrapure water and used as subphase in the Langmuir film experiments. Ultrapure water (resistivity of 18.2 MΩ cm) was obtained from a Milli-Q purification system (model Direct-Q 3UV). Nanodiamonds functionalized with carboxyl groups (NDCOOHs) were purchased from ITC–International Technology Center (Durham, NT, USA, Standard Nanodiamond). The colloidal NDs suspension was prepared by dispersing the NDCOOHs in ultrapure water at a concentration of 100 mg/mL, followed by 10 min ultrasonication and 1 min vortexing to improve nanoparticle dispersion and prevent aggregation, obtaining NDCOOHs with approximately 100 nm.^11^

### Langmuir Monolayers of **E. coli** and *S. aureus*: Fabrication and Characterization

Langmuir monolayers were prepared using a KSV Nima Langmuir trough (model 2001) equipped with a platinum Wilhelmy plate sensor. The trough contained a subphase volume of 50 mL, and the subphase temperature was maintained at 21 °C using an ultrathermostatic bath (SolidShell SSDu-10 L). Surface pressure–area (π–*A*) isotherms were recorded for **E. coli** lipid extract and *S. aureus* lipid mixture by spreading their chloroform solutions onto the air-PBS interface. The lipid monolayers of **E. coli** and *S. aureus* were studied both in the absence and presence of NDCOOHs, which were cospread at volume ratios (lipid solution: NDCOOHs) of 1:1, 1:2, and 1:4. After spreading, a 10 min waiting period was allowed to ensure complete evaporation of chloroform. Subsequently, the monolayers were symmetrically compressed at a constant speed of 5 mm/min. Reproducibility of the π–*A* isotherms was verified by ensuring a variability within ±2 mN/m.

Relative area shifts were determined from the π–*A* isotherms using the formula [(*A* – *A*_0_)/*A*_0_] × 100, where *A*_0_ is the extrapolated area at a surface pressure of 30 mN/m for the lipid monolayers of **E. coli** and *S. aureus* without NDCOOHs, and *A* corresponds to the area with NDCOOHs. The surface compression modulus (*C*_s_^–1^) was calculated to evaluate changes in monolayer elasticity induced by the incorporation of NDCOOHs, using *C*_s_^–1^ = −*A*(∂π/∂*A*), where *A* is the area occupied by the monolayer. The stability of the **E. coli** and *S. aureus* lipid monolayers, both with and without NDCOOHs, was examined by maintaining the surface pressure at 30 mN/m and monitoring the changes in relative area over a period of 100 min. Polarization-modulated infrared reflection–absorption spectroscopy (PM-IRRAS) was carried out using a KSV PMI550 Langmuir trough, with an incidence angle of 81° and a resolution of 8 cm^–1^. PM-IRRAS spectra were recorded for the lipid monolayers of **E. coli** and *S. aureus*, both in the absence and presence of cospread NDCOOHs, at a constant surface pressure of 30 mN/m. Reproducibility was ensured to confirm that observed changes in the PM-IRRAS spectra were attributable to the incorporation of NDCOOHs. All experiments were conducted in triplicate.

## Results and Discussion

### NDCOOHs Incorporation into **E. coli** and *S. aureus* Lipid Monolayers

**Figure 2:**
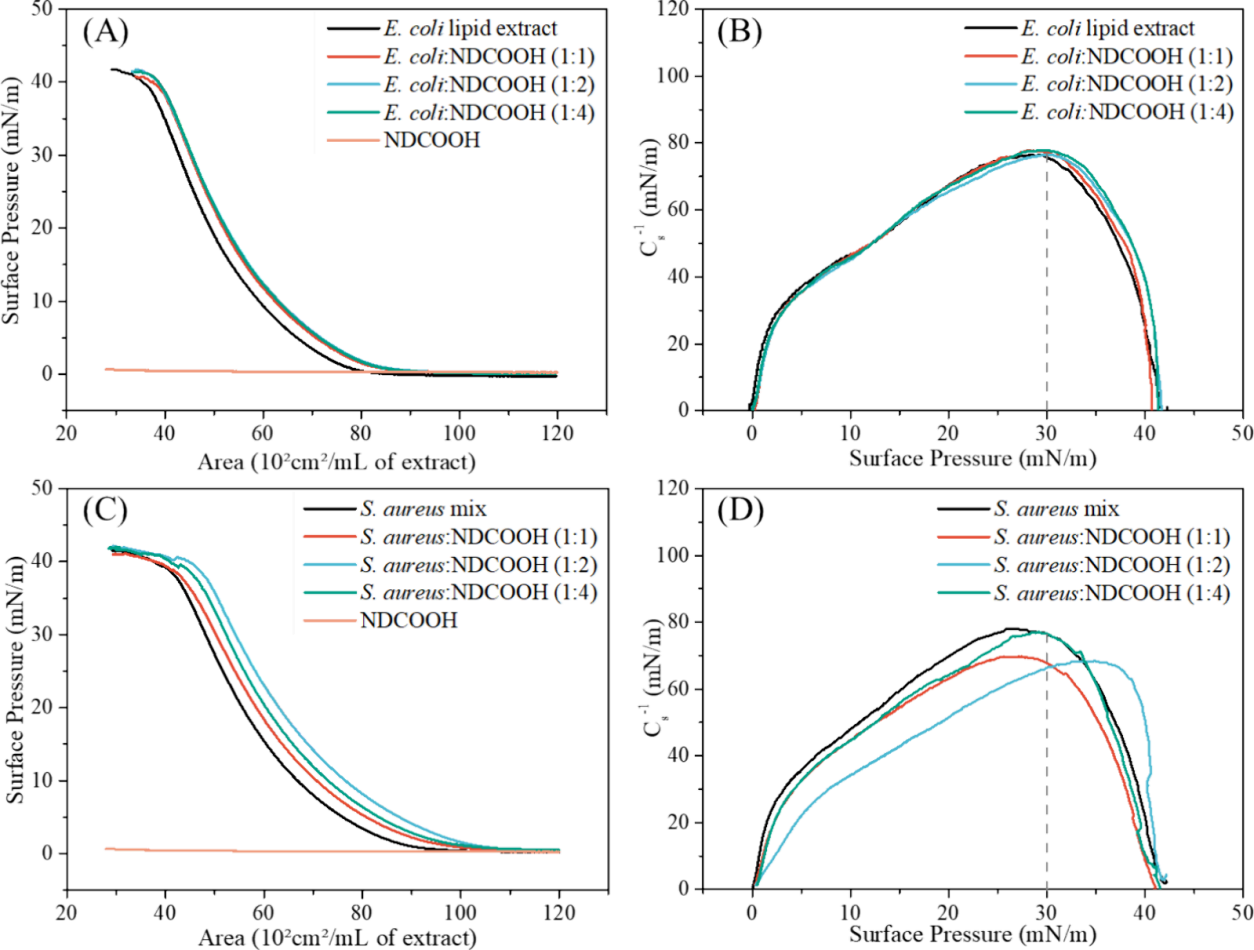
Surface pressure (mN/m) versus area (10^2^ cm^2^/mL lipid extract) isotherms and surface compression modulus (*C*_s_^–1^) for (A) **E. coli** and (B) **E. coli**:NDCOOHs (1:1, 1:2, and 1:4, *v/v*) and for (C) *S. aureus* and (D) *S. aureus*:NDCOOHs monolayers (1:1, 1:2, and 1:4, *v/v*).

The π–*A* isotherms and the surface compression modulus (*C*_S_^–1^) of **E. coli** lipid extract and **E. coli**:NDCOOHs (1:1, 1:2, 1:4 v/v) are shown in Figure 2a,b, while the results for *S. aureus* lipid mixture and *S. aureus*:NDCOOHs (1:1, 1:2, and 1:4 v/v) are displayed in Figure 2c,d, respectively. NDCOOHs on PBS subphase do not exhibit surface activity, suggesting that the expansions in the isotherms arise from the incorporation of NDCOOHs into the lipid monolayers. The incorporation of NDCOOHs into **E. coli** monolayers led to statistically significant (*p* < 0.05) increase in the extrapolated area from 61.5 × 10^2^ to 65 × 10^2^ cm^2^/mL (Table S1), causing displacements in the π–*A* isotherms toward higher area values (Figure 2a). The relative areas shifts for 1:1, 1:2, and 1:4 (**E. coli**:NDCOOHs) were 5.8% ± 1.6%, 7.1% ± 0.7%, and 5.7% ± 1.0%, respectively. No significant differences (*p* > 0.05) were detected between these ratios, indicating limited NDCOOHs incorporation into **E. coli** monolayers. The *C*_s_^–1^ value of the **E. coli** lipid extract monolayer at 30 mN/m (*C*_s_^–1^ = 75.6 mN/m) did not differ significantly from those of the cospread **E. coli**:NDCOOHs monolayers at any volume ratio (Figure 2b and Table S1), indicating that the incorporation of NDCOOHs does not alter the membrane elasticity.), indicating that the incorporation of NDCOOHs does not alter the membrane elasticity. The observed *C*_s_^–1^ are characteristic of a phase transition from the liquid-condensed to the liquid-expanded.^52^ Although the extract consists largely of zwitterionic lipids (57.5% PE), electrostatic repulsion likely occurs between the negatively charged lipids (15.1% PG and 17.6% CL)^53^ and the negatively charged NDCOOHs (zeta potential: −29.21 mV). This repulsion may have restricted further incorporation of NDCOOHs into the **E. coli** lipid extract monolayer. In contrast, the incorporation of the photosensitizer eosin decyl ester (EosDec) into **E. coli** lipid extract monolayers at 10:1 and 5:1 (**E. coli**:EosDec) volume ratios led to more pronounced relative area shifts, viz. 12.5 and 33.9%, respectively.^37^ These displacements were attributed to extensive interactions between EosDec and the lipid carbonyl groups, facilitated by the hydrophobic nature of the photosensitizer.^37^ Despite the electrostatic repulsion between NDCOOHs and the negatively charged lipids (PG and CL) in the **E. coli** extract, monolayer alterations were observed, likely due to interactions with the zwitterionic lipids (57.5% PE). Further insights into these interactions will be explored through PM-IRRAS analysis.

*S. aureus* lipid monolayers had an initial extrapolated area of 72.2 × 10^2^ cm^2^/mL. With the incorporation of NDCOOHs, this increased to 74.7, 80.3, and 76.1 × 10^2^ cm^2^/mL for *S. aureus* at volume ratios of 1:1, 1:2, and 1:4, respectively (Table S1). These changes correspond to relative area shifts of 3.5% ± 2.4% (1:1), 11.1% ± 2.5% (1:2), and 5.4% ± 3.8% (1:4), indicating the highest incorporation of NDCOOHs at the 1:2 ratio (Figure 2c and Table S1). This may be due to the presence of 15% LPG, a positively charged lipid, in the *S. aureus* mixture,^34^ which could favor NDCOOHs binding at the–NH_3_^+^ cationic groups on the lipid polar heads.^36,54^ The reduced relative area shift at the 1:4 ratio, showing no significant difference (*p* > 0.05) compared to 1:1, further supports this pattern (Table S1). Additionally, the presence of Na^+^ counterions around DOPG and CL may reduce repulsive interactions with NDCOOHs, promoting their incorporation through secondary interactions.^36^ Incorporating NDCOOHs at 1:1 and 1:2 volume ratios reduced the *C*_s_^–1^ values of the *S. aureus* monolayers at 30 mN/m, from 76.4 to 67.7 mN/m and 66.3 mN/m, respectively (Figure 2d and Table S1), indicating increased monolayer flexibility^37,52^ at these ratios. While the *C*_s_^–1^ values for 1:1 and 1:2 are comparable, the distinct curve profile at the 1:2 ratio supports the higher incorporation of NDCOOHs observed in the π–*A* isotherms, suggesting the most significant effect of NDCOOHs on the *S. aureus* monolayer. Changes in membrane elasticity are important, as the effectiveness of nanoparticles often depends on their ability to alter membrane flexibility, thereby facilitating mass transport or even compromising membrane integrity.^55^

The effects of nanodiamonds on individual lipid components may be analyzed in a more straightforward manner and related to the specific properties of each lipid species. Chakraborty et al. reported a condensing effect from anionic nanodiamonds on zwitterionic monolayers of DPPC, reducing the molecular area occupied by the lipid molecules, changing lipid domain morphology, and enhancing lipid packing. In contrast, minimal effects were noted on anionic monolayers of DPPG, with only a slight increase in fluidity. Such effects suggest that incorporation of anionic nanodiamonds into individual lipid monolayers may depend on electrostatic interactions with the lipid charged groups.^47^ In lipid mixture monolayers, the effects of nanodiamonds may differ from individual lipid components. Indeed, the incorporation of nanodiamonds increased slightly the monolayer area of zwitterionic-rich monolayer of **E. coli** lipid extract (PE > PG > CL) and did not alter the elasticity. However, for the anionic-rich monolayer of *S. aureus* lipid mixture (DOPG > LPG > LTA > CL), a stronger incorporation of NDCOOHs led to a significant increase in monolayer area and flexibility. Therefore, both the charge of the lipid headgroups and the saturation of the lipid alkyl chains may be involved in the electrostatic and secondary interactions with nanodiamonds.^47^

**Figure 3:**
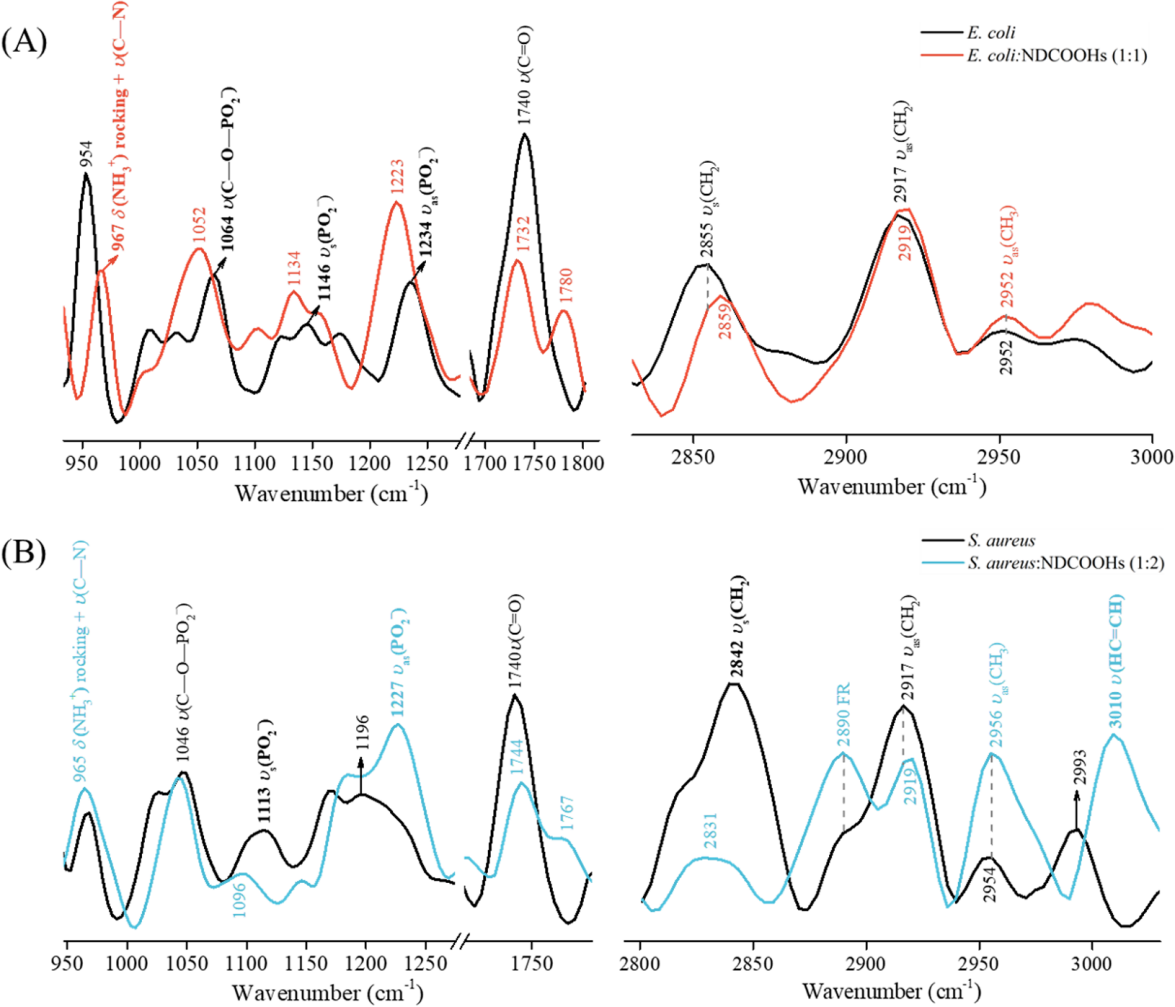
PM-IRRAS spectra for (A) **E. coli** and **E. coli**:NDCOOHs (1:1) and (B) *S. aureus* and *S. aureus*:NDCOOHs (1:2) monolayers on the PBS subphase at 30 mN/m. The left panels highlight bands related to polar head groups, while the right panels refer to those associated with alkyl chain groups.

Figure 3a,b presents the PM-IRRAS spectra for **E. coli** and *S. aureus* lipid monolayers cospread with NDCOOHs, at 1:1 (**E. coli**) and 1:2 (*S. aureus*) ratios, respectively. These ratios were selected based on their significant effects observed in the isotherms, specifically in relative area shift and surface compression modulus. Table 1 summarizes the assignments of the main vibrational modes along with the modifications induced by NDCOOHs incorporation. This incorporation had a greater impact on the polar head groups of the **E. coli** lipid extract monolayer than on the aliphatic chains. For instance, the δ(NH_3_^+^) rocking + υ(C–N)^36^ at 954 cm^–1^ shifted to 967 cm^–1^, indicating attractive electrostatic interactions between the ammonia groups (−NH_3_^+^) from PE (57.5% of **E. coli** lipid extract composition) and the negatively charged NDCOOHs.^11,23^ The effects from NDCOOHs were also significant in the phosphate groups, as the bands ν(C–O–PO_2_^–^)^39^ at 1064 cm^–1^, ν_s_(PO_2_^–^)^36^ at 1146 cm^–1^, and ν_as_(PO_2_^–^)^38^ at 1234 cm^–1^ displaced to 1052, 1134, and 1223 cm^–1^, respectively. More specifically, the shift of ν_as_(PO_2_^–^) to lower wavenumbers indicates hydration of the phosphate groups in the monolayer upon NDCOOHs incorporation.^56^ Changes in the vibrational modes of phosphates suggest repulsive electrostatic interactions with the negatively charged NDCOOHs, which is consistent with the composition of the **E. coli** lipid extract, where phosphate-containing lipids make up 57.5% PE, 15.1% PG, and 9.8% CL. The band at 1740 cm^–1^, assigned to the nonhydrated ν(C=O),^57^ shifted to 1732 cm^–1^ with a decrease in relative intensity. Besides, a new band appeared at 1780 cm^–1^, also corresponding to ν(C=O), but from the NDCOOHs. According to Tu et al.^58^ this vibrational mode refers to the functionalized −COOH groups and depends on the size of nanodiamonds, shifting from 1680 to 1820 cm^–1^ as the nanodiamond diameter increases from 5 to 500 nm.^58^ For nanodiamonds around 100 nm, which is the size of the NDCOOHs studied here, ν(C=O) is expected between 1760 and 1775 cm^–1^, consistent with our results. These findings, along with the changes observed in the phosphate regions, indicate that NDCOOH incorporation affects the phosphate region and extends to the carbonyl ester groups of the **E. coli** lipid extract monolayer.

**Table 1:** Assignments of the Main Vibrational Modes of **E. coli** and *S. aureus* Monolayers along with the Shifts Induced by NDCOOHs Incorporation

	**E. coli** (cm^–1^)		*S. aureus* (cm^–1^)		references
assignments	PBS	NDCOOHs	PBS	NDCOOHs	
δ(NH_3_^+^) rocking + υ(C–N)	953	967	965	965	(36)
ν(C–O–PO_2_^–^)	1052	1064	1046	1047	(39)
ν_s_(PO_2_^–^)	1146	1134	1113	1096	(36)
ν_as_(PO_2_^–^)	1234	1223	1196	1227	(38)
ν(C=O)	1740	1732	1740	1744	(57)
ν_s_(CH_2_)	2855	2859	2842	2831	(59)
FR			2890	2890	(65)
ν_as_(CH_2_)	2917	2919	2917	2919	(59)
ν_as_(CH_3_)	2952	2952	2954	2956	(38)
ν(HC=CH)			2993	3010	(36)

The incorporation of NDCOOHs induced minimal changes in the alkyl chain bands, as shown in Figure 3a (right panel). The ν_s_(CH_2_)^59^ shifted slightly from 2855 to 2859 cm^–1^, while the intensity ratio of ν_s_(CH_2_) to ν_as_(CH_2_) (I_s_/I_as_) decreased from 0.64 to 0.40, suggesting increased ordering of hydrocarbon chains.^60,61^ Jaroque et al.^59^ demonstrated that interactions between cytosporone-B, a polyketide with antimicrobial properties, and the all-trans conformation of alkyl chains can increase the I_s_/I_as_ ratio by promoting a more ordered state through van der Waals forces. Similarly, this mechanism could be extended to explain van der Waals interactions between NDCOOHs and the hydrocarbon chains of the **E. coli** monolayer. Therefore, NDCOOH incorporation into the **E. coli** lipid monolayer is primarily driven by electrostatic interactions with polar head groups. Attraction from ammonia (−NH_3_^+^) groups facilitates adsorption, while repulsion from phosphate and carbonyl ester groups restricts deeper penetration, influencing only minimally the alkyl chain ordering.

For *S. aureus* monolayer, the bands attributed to the δ(NH_3_^+^) rocking + ν(C–N) and ν(C–O–PO_2_^–^) were not altered, as shown in Figure 3b (left panel). On the other hand, ν_s_(PO_2_^–^) shifted from 1113 to 1096 cm^–1^ and ν_as_(PO_2_^–^) shifted from 1196 to 1227 cm^–1^, with an increase in relative intensity. Unlike for the **E. coli** monolayer, the insertion of NDCOOHs into *S. aureus* monolayers caused a shift in the ν_as_(PO_2_^–^) band to higher wavenumbers. This indicates that the interaction with NDCOOHs affected the hydrogen bonds with surrounding water,^62,63^ thereby causing dehydration of the phosphate groups.^38,64^ With the *S. aureus* lipid mixture containing 75% DOPG, 15% LPG, and 3% CL, the negative charge of NDCOOHs likely causes the changes in phosphate vibrational modes through repulsive electrostatic interactions. The nonhydrated ν(C=O)^57^ at 1740 cm^–1^ shifted to 1744 cm^–1^ with a reduction in relative intensity. Additionally, a band at 1767 cm^–1^ emerged upon NDCOOHs incorporation, attributed to ν(C=O) from the functionalized groups of NDCOOHs.^58^ These changes suggest that NDCOOHs incorporation has a greater impact on the phosphate region than on the carbonyl ester groups of the *S. aureus* lipid monolayer. As for the alkyl chain groups in the right panel in Figure 3a, the effects from NDCOOHs incorporation are more pronounced than in the **E. coli** monolayer. For instance, ν_s_(CH_2_)^59^ shifted from 2842 to 2831 cm^–1^, with a decrease in intensity. The Fermi resonance (FR) band at 2980 cm^–1^, observed only in the spectra of *S. aureus* monolayer alkyl chains, arises from lateral interchain interactions between neighboring methylene (−CH_2_) groups.^65^ Meanwhile, ν_as_(CH_3_)^39^ at 2954 cm^–1^ decreased in intensity, and the intensity ratio of ν_s_(CH_2_) to ν_as_(CH_2_) bands (I_s_/I_as_) decreased from 1.10 to 0.32. Additionally, the ν(HC=CH)^36^ shifted from 2993 to 3010 cm^–1^, with increased intensity. These changes suggest that NDCOOHs insertion into the *S. aureus* monolayer increases the conformational order of the alkyl chains. Indeed, in a PBS subphase (pH 7.4), the dominant deprotonated^58,66^ NDCOO^–^ species enable secondary interactions with membrane lipids, alongside electrostatic forces. The NDCOO^–^ groups also increase the potential for hydrogen bonding.^58^

**Figure 4:**
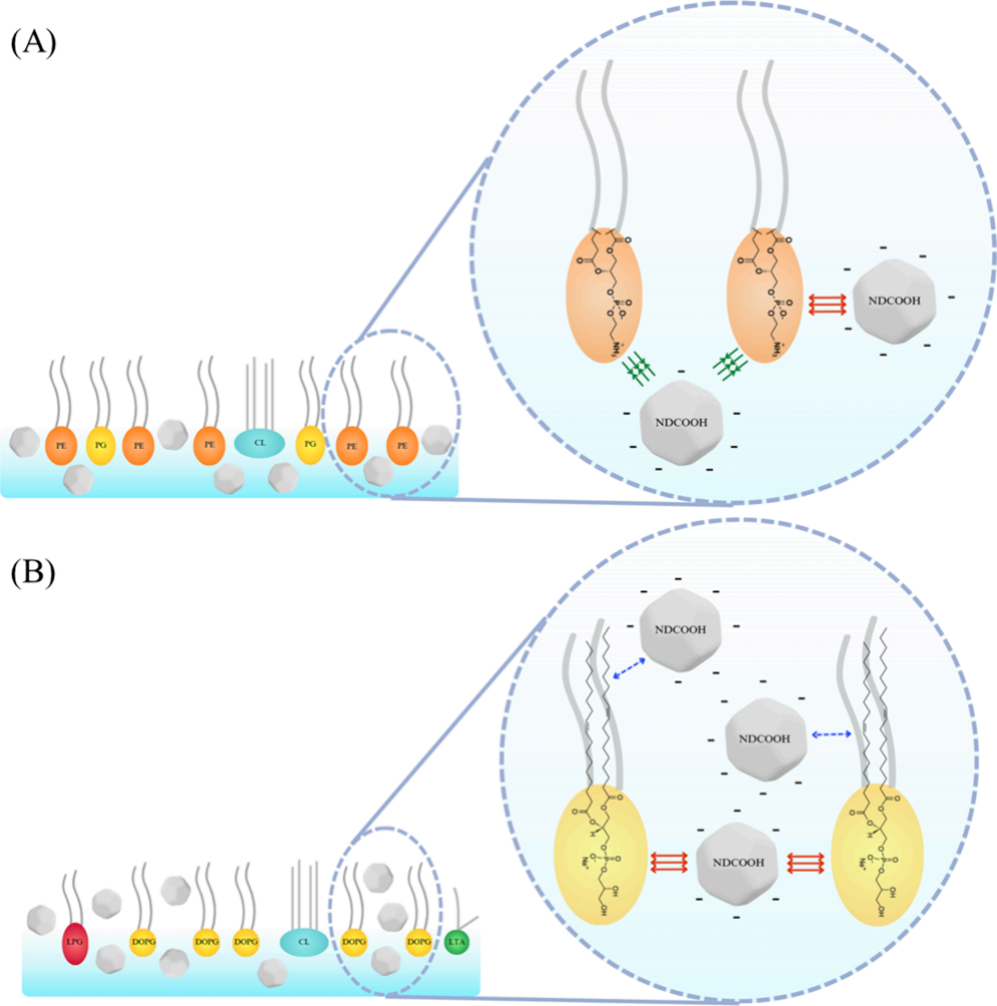
Schematic representation of the binding mechanism of NDCOOHs in (A) **E. coli** extract and (B) *S. aureus* lipid mixture monolayers. Green arrows indicate attractive electrostatic interactions, red arrows show repulsive electrostatic interactions, and blue dashed arrows illustrate secondary interactions, such as van der Waals and hydrogen bonding.

Overall, deeper penetration of NDCOOHs into *S. aureus* monolayers likely led to more pronounced changes in the hydrocarbon chains compared to **E. coli** monolayers. Attractive electrostatic interactions with the polar head groups of **E. coli**, likely the ammonia groups, may have confined NDCOOHs to the lipid head region, preventing access to the chain region. In contrast, the greater modifications in the alkyl chains of *S. aureus* monolayers indicate that more NDCOOHs are inserted, particularly in the tails, increasing the surface area occupied by the monolayer while maintaining a more ordered conformation of the chains. An illustrative model of the binding mechanism is presented in Figure 4a,b. These modifications in the model systems could contribute to explain the differential effects observed in vitro, as reported in the work of Ong et al.,^29^ wherein the partially oxidized monocrystalline nanodiamonds exhibited both bactericidal and bacteriostatic against *S. aureus* whereas they showed minimal impact against **E. coli**. Notably, antimicrobial activity can change with NDs concentration. Since the NDs showed better interaction into the lipid bilayer of *S. aureus* compared with the interaction into **E. coli**, it is possible to infer that NDs could internalize into **E. coli** instead of staying dispersed through the bilayer. Therefore, as reported by Hurtado et al.^11^ depending on the concentration, the presence of NDs inside the bacteria compromise their viability and NDs would display higher antibacterial activity.

**Figure 5:**
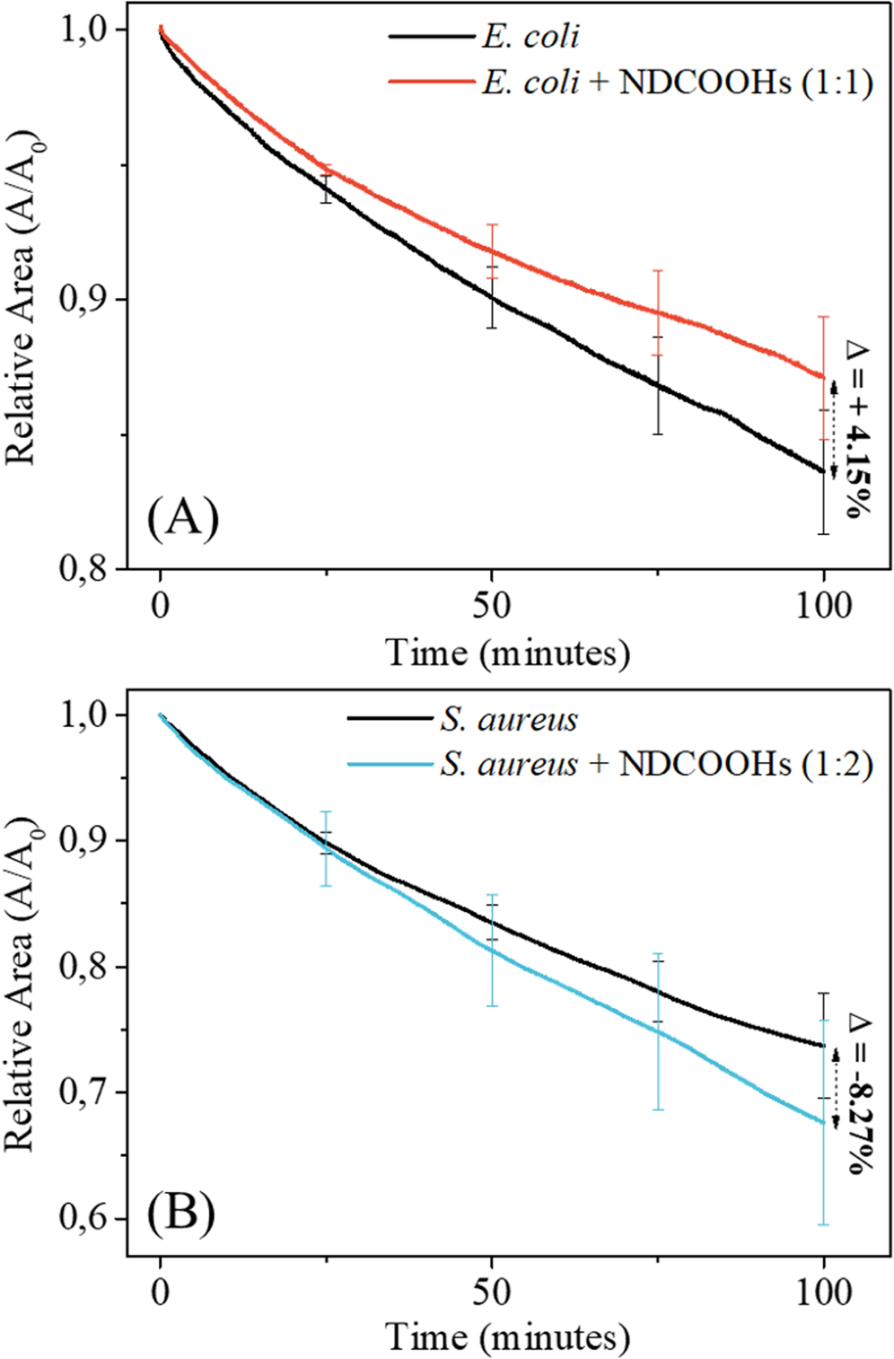
Comparative evolution of relative area (*A*/*A*_0_) versus time (100 min) for (A) **E. coli** and **E. coli**:NDCOOHs (1:1) and for (B) *S. aureus* and *S. aureus*:NDCOOHs (1:2) recorded at constant pressure of 30 mN/m. *A*_0_ is the extrapolated area of the isotherms at 30 mN/m down to zero pressure.

The impact of NDCOOHs incorporation on **E. coli** and *S. aureus* monolayers was also assessed by monitoring the evolution of relative surface area in the absence and presence of NDCOOHs, at a constant pressure of 30 mN/m. Figure 5a,b depicts the relative area evolution (*A*/*A*_0_) versus time (min) for **E. coli** and **E. coli**:NDCOOHs (1:1), and *S. aureus* and *S. aureus*:NDCOOHs (1:2), respectively. The decrease in the relative area of the monolayers in the absence of NDCOOHs (with only PBS as subphase) can be attributed to uncontrolled oxidation mediated by reactive oxygen species (ROS) in the environment, which results in the loss of material to the subphase.^67^ The reduction in relative area for *S. aureus* (26.29%) and *S. aureus*:NDCOOHs (32.38%) monolayers (Figure 5b) was more pronounced than that for **E. coli** (16.39%) and **E. coli**:NDCOOHs (12.92%) monolayers (Figure 5a). The *S. aureus* lipid mixture consists entirely of unsaturated lipids, whereas the **E. coli** lipid extract includes an undetermined percentage of unsaturated lipids in PE and PG. According to Liljeblad et al.^67^ unsaturated lipids are more unstable to air exposure than saturated lipids, which could explain the differing effects on the reduction in relative area for *S. aureus* and **E. coli** monolayers.

The incorporation of NDCOOHs resulted in ca. 4.15% increase in the relative area of the **E. coli**:NDCOOHs (1:1) compared to the **E. coli** monolayer (Figure 5a). In contrast, the *S. aureus*:NDCOOHs (1:2) showed an ca. 8.27% decrease in relative area compared to the *S. aureus* monolayer (Figure 5b). Although these variations are not statistically significant (*p* > 0.05), the tendency for the relative area to increase or decrease can be interpreted as an indication of the effects of NDCOOHs incorporation. Shieh et al.^68^ associated antibacterial effects of oxidized nanoparticles to electronic changes in local structures. According to Chatterjee et al.*,*^9^ these electronic effects cause electrons to react with molecular oxygen (O_2_) forming superoxide radicals, while the holes react with the surface OH groups to form hydroxyl (OH) radicals. These radicals attack organic molecules, which are eventually oxidized, thus achieving an antibacterial effect.^9,40^ Therefore, since NDCOOHs are functionalized with carboxylic acids, it can be hypothesized that superoxide and/or hydroxyl radicals are being formed, oxidizing the lipids of the **E. coli** and *S. aureus* monolayers. NDCOOHs incorporation may trigger oxidative reactions that result in chain cleavage of *S. aureus* lipid chains, leading to membrane damage, material loss to the subphase, and consequently a decrease in surface area.^34,36,37,40^ In contrast, the limited penetration of NDCOOHs into **E. coli** monolayer may have triggered the generation of lipid hydroperoxide groups at the unsaturation sites, affecting the hydrophilic–hydrophobic balance of the membrane and expanding the surface area.^34^ These results suggest that the mechanisms of NDCOOHs incorporation may depend on membrane composition and that interactions with the bacterial cell membrane could play a role in their antibacterial action.

## Conclusions

The effects from NDCOOHs on lipid monolayers of *E. coli* and *S. aureus* have been investigated, focusing on the role of molecular-level interactions in modulating membrane properties. The area of **E. coli** monolayers increased upon NDCOOH addition (from 61.5 to 65 × 10^2^ cm^2^/mL); however, the lack of statistical significance between volumetric ratios suggests limited incorporation. Similarly, the unchanged *C*_s_^–1^ values indicate that NDCOOHs incorporation does not affect the flexibility of **E. coli** monolayers. In contrast, a higher NDCOOHs incorporation was observed for *S. aureus* monolayers, with area increase from 72.2 to 80.3 × 10^2^ cm^2^/mL at a 1:2 volume ratio. At this ratio, *C*_s_^–1^ values decreased from 76.4 to 66.3 mN/m, indicating increased monolayer flexibility post-NDCOOH addition. PM-IRRAS analysis revealed significant impacts of NDCOOH incorporation on the polar head groups of **E. coli** monolayers, with electrostatic attraction to ammonium groups and repulsion from phosphate and carbonyl ester groups, which likely restrict deeper NDCOOH penetration into lipid chains. Conversely, *S. aureus* monolayers showed pronounced alterations in the aliphatic hydrocarbon chains, suggesting deeper NDCOOH penetration facilitated by favorable secondary interactions. The stability of **E. coli** and *S. aureus* monolayers was also affected, with NDCOOHs increasing the relative area of the **E. coli** monolayer by 4.15% and reducing that of *S. aureus* by approximately 8.27%. While these changes were not statistically significant, they suggest a potential for superoxide or hydroxyl radical generation, possibly leading to lipid oxidation. In summary, these findings reveal key interactions between carboxyl-functionalized nanodiamonds and bacterial membrane models, offering a foundation for understanding complex in vitro systems and advancing antimicrobial nanomaterials.

## Supporting Information Available

Extrapolated areas derived from surface pressure (π)–area (*A*) isotherms, relative area shifts, and surface compression modulus and FTIR vibrational modes of the NDCOOHs drop-cast film (PDF)
