# Supplementary material for: Determining Molecular-Level Interactions of Carboxyl-Functionalized Nanodiamonds with Bacterial Membrane Models as the Basis for Antimicrobial Activity
Source: Langmuir. 2025 Mar 2;41(9):6186–96. doi: 10.1021/acs.langmuir.4c05173 (PMC11912534; doi:10.1021/acs.langmuir.4c05173)
Supplement: Supplementary file 1 — la4c05173_si_001.pdf [file la4c05173_si_001.pdf]

Supporting Information

Determining Molecular-Level Interactions of Carboxyl-Functionalized Nanodiamonds with Bacterial Membrane Models as the Basis for Antimicrobial Activity

Giovanna Eller Silva Sousa[1], Bruna Alves Martins[1], Alexandre Mendes de Almeida Junior[1], Rafaela Campos Queiroz[2], Dayane Batista Tada[2], Sabrina Aléssio Camacho[1], Osvaldo Novais Oliveira Junior[3], Pedro Henrique Benites Aoki[1]*

[1] São Paulo State University (UNESP), School of Sciences, Humanities and Languages, Assis, SP, 19806-900, Brazil

[2] Federal University of São Paulo (UNIFESP), Institute of Science and Technology, São José dos Campos, SP, 12231-280, Brazil.

[3] University of Sao Paulo (USP), São Carlos Institute of Physics, São Carlos, SP, 13566-590, Brazil

*Corresponding author: pedro.aoki@unesp.br

Contents:

Table S1. Extrapolated area, relative area shift, and surface compression modulus for the π-A isotherms of *E. coli* and *E. coli*:NDCOOHs (1:1, 1:2, and 1:4 v/v), *S. aureus* and *S. aureus*:NDCOOHs (1:1, 1:2, and 1:4 v/v), at surface pressure of 30 mN/m.

S-2

Figure S1. FTIR spectrum of the NDCOOHs drop-cast film.

S-3

Contents of the Supporting Information

Number of pages: 3

Number of Tables: 1

Number of Figures: 1

Table S1. Extrapolated area, relative area shift, and surface compression modulus for the π-A isotherms of *E. coli* and *E. coli*:NDCOOHs (1:1, 1:2, and 1:4 v/v), *S. aureus* and *S. aureus*:NDCOOHs (1:1, 1:2, and 1:4 v/v), at surface pressure of 30 mN/m.

	**Extrapolated area (10^2 cm^2/mL of lipid extract)**	**Relative area shift (%)**	**Surface compression modulus (mN/m)**
E. coli lipid extract	61.5 ± 0.3 ^{a}	-	75.6
E. coli:NDCOOHs (1:1)	65.1 ± 0.8 ^{b}	5.8 ± 1.6 ^{a}	77.4
E. coli:NDCOOHs (1:2)	65.9 ± 0.4 ^{b}	7.1 ± 0.7 ^{a}	76.5
E. coli:NDCOOHs (1:4)	65.0 ± 0.9 ^{b}	5.7 ± 1% ^{a}	77.8
S. aureus mix	72.2 ± 0.6 ^{a}	-	76.4
S. aureus:NDCOOHs (1:1)	74.7 ± 1.7 ^{a}	3.5 ± 2.4 ^{a}	67.7
S. aureus:NDCOOHs (1:2)	80.3 ± 1.9 ^{b}	11.2 ± 2.6 ^{b}	66.3
S. aureus:NDCOOHs (1:4)	76.1 ± 2.8 ^{ab}	5.4 ± 3.8 ^{ab}	76.6

* Statistical differences are represented in such a way that different lowercase letters within the lines indicates a statistical difference by the Tukey test ($p < 0.05$).

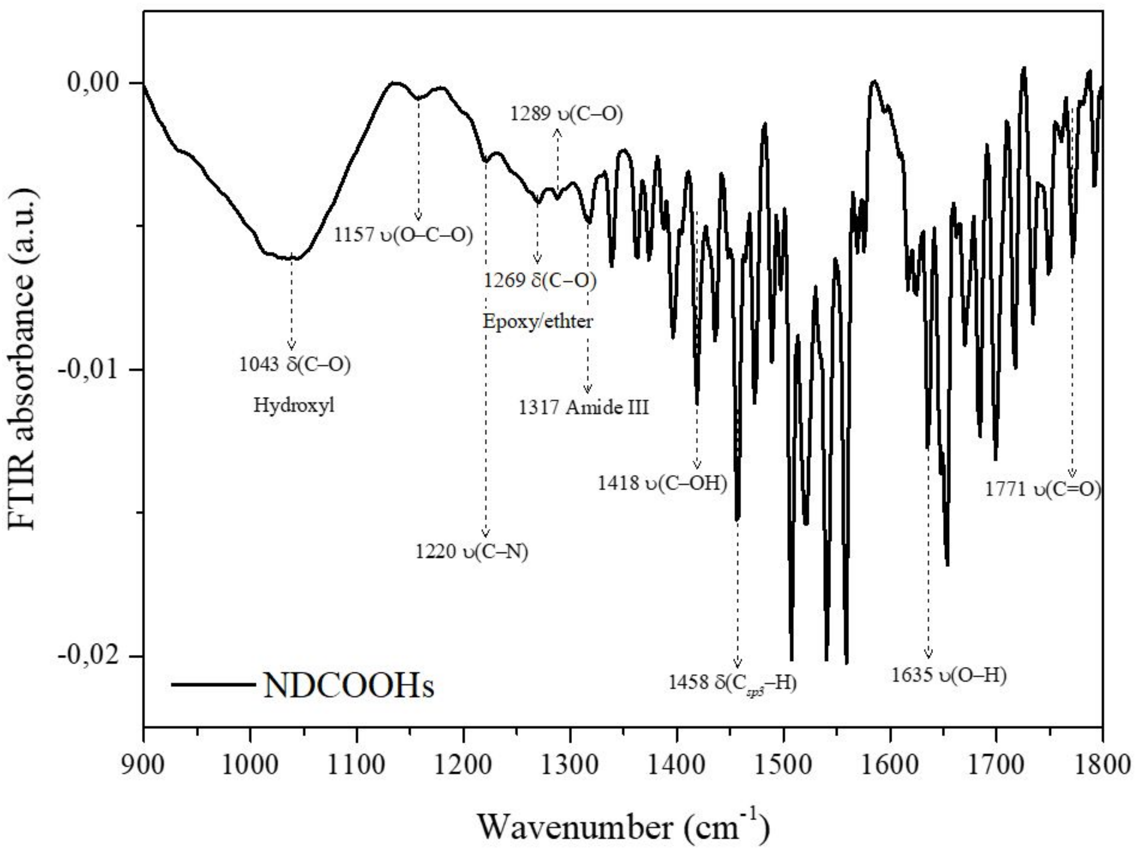

Figure S1. FTIR spectrum of the NDCOOHs drop-cast film.
